# Return to performance: machine learning insights into how absence time following muscle injuries affects match running performance in LaLiga soccer players

**DOI:** 10.5114/biolsport.2025.151651

**Published:** 2025-06-24

**Authors:** Javier Pecci, Horacio Sánchez-Trigo, David Mancha-Triguero, Borja Sañudo, Gonzalo Reverte-Pagola, Juan José del Ojo-López, Roberto López del Campo, Ricardo Resta, Adrián Feria-Madueño

**Affiliations:** 1 Department of Physical Education and Sport, University of Seville, Seville, Spain; 2 University CEU Fernando III, CEU Universities, Spain; 3 Sevilla Football Club, Seville, Spain; 4 Department of Competitions and Mediacoach, LaLiga, Madrid, Spain

**Keywords:** Return to sport, Adductors, Quadriceps, Calf, Hamstrings

## Abstract

To determine how absence time after muscle injuries affects external load metrics in elite soccer players and identify which performance variables are most impacted by the injury. A total of 110 lower limb muscle injuries from LaLiga players were analysed. Following an analysis of pre- and post-injury data to identify which outcomes were affected by muscle injury, machine learning algorithms were employed to examine relationships between absence duration and performance metrics. Maximal speed, maximal acceleration, maximal deceleration, composite index (i.e., overall player performance) and sprint count during matches were the most affected variables after return to play. The multiple linear regression (MLR) model and random forest regression (RFR) presented an R^2^ of 0.348 and 0.442. Maximal speed was the variable most strongly associated with absence time in both models (coefficient in MLR = 7.94; mean absolute SHAP value in RFR model = 4.99), with longer recovery periods correlating with reduced match performance in this metric. Maximal acceleration and deceleration also showed declines with increased absence time. In contrast, sprint count exhibited no significant relationship with absence time. Maximal speed, acceleration and deceleration capacity, as well as sprint count and overall performance, are affected after muscle injuries. However, prolonged recovery following muscle injuries especially reduces maximum speed and acceleration/deceleration capacity in elite players during matches, while sprinting actions remain unaffected by absence time.

## INTRODUCTION

One of the main goals of sport science and health research is identifying independent variables (e.g., injured muscle group) that affect a dependent variable (e.g., running performance), since such information is useful for establishing predictive relationships [1–3]. Traditionally, health behaviour research has depended on regression modelling [4]. However, interpreting these interactions can be challenging, particularly when three or more independent variables are considered [4]. A more contemporary approach to this research issue involves employing machine learning algorithms [5–7]. In this regard, some authors have highlighted that machine learning represents a promising supplementary method to conventional analyses in sports injury and rehabilitation research [8], offering potential for practical research and clinical application in both primary and secondary prevention [2]. The primary concept of machine learning is to create a predictive algorithm (or model) by training on a “labelled” dataset [5, 9]. A variety of statistical techniques are employed to evaluate the significance of the effects of independent variables on the dependent variable [10]. Several machine learning models have been used in the sport science field and in the sports injury field in particular [3, 11, 12]. A systematic review and meta-analysis showed that external load metrics, as well as internal load parameters, are associated with injury risk in professional soccer players based on machine learning models existing in the scientific literature [12]. Nonetheless, when the injury occurs, there are no previous studies using machine learning approaches explaining the changes in key performance metrics through different injury types (e.g., different absence time, involved tissue). This is especially important in muscle injuries given the variability of absence times [13–15]. Return to play following muscle injuries depends on the dimension of the injury as well as the affected tissue, with more tendon involvement usually leading to longer periods of recovery [15–18].

Despite a reduction in overall soccer injury rates in recent years, muscle injury rates have remained constant [19]. This is even more concerning given that muscle injuries present a high recurrence rate [20–23], with longer associated absence periods [20, 22]. Specifically, the hamstrings are the most injured muscle group [22, 23], with one systematic review highlighting that the recurrence rate could be up to 68% [24]. Some studies have shown the impact of injuries in different cohorts, with one study showing significant reductions in playing time, jogging and running distances following injuries without specifying the injury location [25]. Another study [26] also analysed the changes in external load parameters after all-type injuries, showing a significant decrease in maximum speed reached during matches. There are only three studies revealing the effects of specific muscle injuries on match running performance. One of them showed significant reductions in distances covered at high intensities and in explosive distance following rectus femoris injuries [27]. The other two studies assessed changes in external load metrics after hamstring injuries, revealing reductions in maximal speed, highspeed running (HSR) distance and sprinting running distance [28, 29]. However, no previous studies have specifically analysed the impact of absence time on this loss of performance, which could be an important variable since the lack of competitive stimulus (e.g., substitutes) is an important factor for decreasing performance [30]. Moreover, the consequences of calf and adductor injuries for match running performance have not been studied. Understanding the key variables that explain running performance decreases could be of interest for practitioners to focus the rehabilitation on those abilities related to the actual decline in performance. Some authors have emphasized this approach, suggesting that rehabilitation should not only aim for return-to-play (i.e., the ability to fully participate in team sessions and competitive matches) but also include a return-to-performance objective. This latter goal involves achieving pre-injury levels in key performance metrics, such as high-intensity actions (e.g., sprinting, HSR) [31, 32]. Consequently, the aim of the present study was to analyse how absence time explains the loss of performance in LaLiga elite soccer players following muscle injuries. Specifically, based on external load parameters and demographical information, this study aimed to analyse the relationship between absence time and loss of performance in the main external load metrics in elite soccer players.

## MATERIALS AND METHODS

### Participants

A total of 110 injuries from 90 male players who competed in the First Division of the Spanish Professional Soccer League (LaLiga) during the season 2022–2023 were collected for this study. Following previous procedures [25], four pre-injury and four post-injury matches were selected for analyses. Those players who did not present pre-injury or post-injury data were discarded. This resulted in 880 match observations for each variable. For inclusion, the injury must have been confirmed in a medical report or at least through the club’s official media. Goalkeepers’ injuries were not considered due to their very different game demands in terms of match running external load [33]. Lower-limb acute muscle injuries were considered for inclusion. If the medical report specified that the injured tissue was the tendon without involvement of the muscle tissue, it was not considered due to the substantially longer absence times for these injuries, as well as due to the very different biomechanical implications [15–17]. LaLiga authorized the use of data regarding match demands for this study, and, in accordance with LaLiga’s ethical guidelines, this investigation does not include any information that identifies individual soccer players.

### Procedures

The impact of muscle injuries on soccer players was analysed through a retrospective design collecting the injuries occurring during the 2022–2023 season. Two authors (JP and DM-T) independently collected the injured players, the date of the injury (i.e., date of official report), the date of return to play (i.e., date of the first match in which the player was available for competing again) and the affected muscle group (i.e., hamstrings, quadriceps, calf or adductors). When a player suffered a re-injury, it was differentiated in the anonymized codes. Then, two authors (AF-M and GR-P) confirmed the data extraction and removed duplicates. Once the information about the injured players was fully collected and confirmed, LaLiga provided data for external load parameters for the four pre-injury matches and the four post-injury matches. Finally, main outcomes were introduced for analyses.

### Main outcomes

The following demographic variables were considered for analyses:

– Injured muscle group: Categorized as 1) hamstrings, 2) adductors, 3) quadriceps, 4) calf, 5) other.
– Main position of the player in the field.
– Changes in position: This was categorized as yes/no. A player was categorized as yes if his position substantially changed throughout the season (e.g., from centre back to full back) due to the demonstrated significant differences in external load demands between positions [34–36].
– Ranked position in the classification of the team in which the player competed.
– Tier (i.e., from 1 to 4, dividing the 20 competing teams into groups of 5 teams) of the team based on the classification during the 2022–2023 season.
– Number of re-injuries represented during the season for each analysed injury.

The following match running variables were considered for analyses for each match, based on previous studies assessing external load metrics from LaLiga players [37–40]:

– Number of accelerations and decelerations, regardless of the intensity (n)
– Total distance covered (m)
– Distance covered accelerating > 3 m/s^2^ (m)
– Distance covered decelerating < -3 m/s^2^ (m)
– Number of absolute HSR (21–24 km/h) actions (n)
– Distance covered at absolute HSR (m)
– Number of relative HSR (> 75.5% of the player’s maximum speed based on the WIMU profile) actions (n)
– Distance covered at relative HSR (m)
– Maximal acceleration registered (m/s^2^)
– Maximal deceleration registered (m/s^2^)
– Maximal speed registered (km/h)
– Distance covered sprinting (> 24 km/h)
– Number of absolute sprints (> 24 km/h) performed
– Number of relative sprints (> 85% of the player’s maximum speed based on the WIMU profile) actions
– Time played (min).

In addition, a composite index based on the acceleration-specific performance (component 1), high-intensity running-related variables (component 2), and medium intensity action variables (component 3) were also considered for analyses [41]. This composite index summarizing the match running performance was calculated following previously established procedures based on three latent components [41]:

1. *Latent component 1*_i_ *= −0.88 × Count of accelerations (zone 2–3 m/s* ^2^ *) − 0.06 × Count of accelerations (zone 3–4 m/s* ^2^ *) − 0.01 × Count of accelerations (zone 4–5 m/s* ^2^ *) + 0.04 × Count of accelerations (zone 5–6 m/s* ^2^ *) + 0.07 × Count of decelerations (zone 2–3 m/s* ^2^ *) + 1.44 × Explosive distance − 0.15 × Count of actions (zone 6–12 km/h)*
2. *Latent component 2*_i_ *= −0.04 × Count of actions (21–24 km/h) + 0.13 × Count of actions (> 24 km/h) + 0.94 × Time spent (zone 21–24 km/h)*
3. *Latent component 3i = 0.10 × Average speed (km/h) − 0.49 × Count of actions (zone 12–18 km/h) + 0.23 × Count of actions (zone 18–21 km/h) − 0.01 × Time spent (zone 18–21 km/h) − 0.04 × Energy expenditure − 0.33 × High-metabolic load actions + 1.11 × High-metabolic load distance*
4. *Raw composite index*_i_ *= 0.29 - Latent component 1*_i_ *+ 0.39 - Latent component 2*_i_ *+ 0.35 - Latent component 3*_i_

### Statistical analysis

For each performance metric, two aggregate variables were created: a pre-injury average and a post-injury average, calculated as the mean across the four pre- and post-injury matches, respectively. A difference variable was also computed to capture the net change between the post- and pre-injury averages for each metric. To determine whether these pre- and post-injury differences were statistically significant, the normality of each parameter’s difference distribution was assessed using the Shapiro-Wilk test, with a significance level of α = 0.05. For metrics where normality was confirmed, a paired t-test was applied to compare pre- and post-injury averages; otherwise, the non-parametric Wilcoxon signed-rank test was used. Only parameters showing statistically significant differences between pre- and post-injury averages were included in subsequent machine learning analyses.

### Machine learning analysis

The aim of this machine learning analysis was to examine the relationship between absence time (days away from competition) and the magnitude of performance changes across parameters that showed statistically significant differences between pre- and post-injury averages. Players with missing data were excluded to ensure complete datasets for analysis. All variables were then scaled to normalize each feature’s distribution. The dataset was divided into training (80%) and testing (20%) subsets via random sampling.

Two regression models were employed to investigate potential relationships between performance changes and absence time: multiple linear regression (MLR) and random forest regression (RFR), supplemented by Shapley Additive exPlanations (SHAP). MLR predicts a single dependent variable based on multiple independent variables through a linear relationship, providing direct interpretability due to its reliance on a linearity assumption [42], since regression coefficients directly reflected each variable’s association with absence time, with larger coefficients indicating stronger relationships. However, MLR is limited in modelling complex, non-linear interactions among features [43]. To capture non-linear relationships, a random forest model was also implemented. RFR, a decision tree-based approach, divides samples into homogeneous groups through successive queries on each variable, thus minimizing within-group variance [44]. Unlike MLR, RFR does not rely on assumptions about data distribution, making it well suited for analysing diverse, complex datasets [45, 46]. However, its “black box” nature can limit interpretability, despite strong predictive power [47]. To address this interpretability challenge, we employed SHAP, a technique that quantifies the contribution of each input variable to the RFR model [48]. SHAP values measure a feature’s importance by comparing the model’s predictions with and without that feature, effectively providing an additive feature attribution method that enhances model interpretability [49, 50]. Thus, in RFR, SHAP values highlight the magnitude and direction of each feature’s relationship with absence time, identifying the performance changes most strongly associated with absence time.

Model performance was evaluated through mean square error (MSE) and the coefficient of determination (R^2^) on the test data [46]. MSE quantified the model’s predictive error, while R^2^ indicated the proportion of variance in absence time explained by the changes in performance variables. A linear regression model was constructed to analyse the relationship between absence time (in days) and changes in the variable showing a stronger association with absence time based on machine learning models (i.e., maximal speed). The absence time was treated as the independent variable, while the maximal speed change served as the dependent variable. Confidence intervals (95%) for the regression line were included to provide an estimate of the precision of the model.

All machine learning analyses were conducted in Python, using libraries such as ‘scikit-learn’ (https://scikit-learn.org/) to streamline data pre-processing, feature selection, and model implementation.

## RESULTS

### Descriptive statistics

The dataset included 110 injuries from players who competed in La Liga, with observations for various performance metrics recorded in both pre- and post-injury matches. Table 1 summarizes key descriptive statistics. The mean number of days players were away from competition due to injury (i.e., absence time) was 34.6 ± 27.9. Muscle injuries were categorized as hamstrings (n = 51), quadriceps (n = 12), calf (n = 18), adductors (n = 12) and other lower limb muscle injuries (n = 17). Across all performance metrics, pre- and post-injury averages were calculated, along with their standard deviations (Table 1).

**TABLE 1:** Descriptive data for pre- and post-muscle injury main external load metrics

	Pre		Post		Difference	

	Mean	SD	Mean	SD	Mean	SD
**Time (min)**	53.9175	29.5772	34.8974	27.1714	-19.0201	31.3393
**Distance (m/min)**	110.3006	10.4556	112.6550	10.3455	1.6318	7.7812
**Acceleration counts per minute**	20.4645	0.9065	20.4764	0.8422	0.0232	0.9441
**Deceleration counts per minute**	20.7663	0.9347	20.7481	0.8110	0.0019	0.9598
**Maximal acceleration (m/s^2^)**	5.1819	0.3536	5.0696	0.4293	-0.1112	0.3995
**Maximal deceleration (m/s^2^)**	-6.0169	0.5051	-5.8640	0.4693	0.1519	0.5624
**Distance (m) accelerating at HI per minute**	5.3440	1.3432	5.4651	1.7657	0.1151	1.2739
**Distance (m) decelerating at HI per minute**	5.7361	1.3849	5.8210	1.6832	0.0236	1.2777
**Count of absolute sprinting actions per minute**	0.2112	0.0836	0.2096	0.0943	-0.0040	0.0664
**Count of relative HSR actions per minute**	0.1498	0.0657	0.1563	0.1011	-0.0061	0.0654
**Count of absolute HSR actions per minute**	0.4799	0.1401	0.4888	0.1598	0.0030	0.1113
**Count of relative sprinting actions per minute**	0.2698	0.3865	0.1782	0.2997	-0.1245	0.4182
**Distance (m) at relative HSR per minute**	3.0692	1.5867	3.2118	2.1837	-0.0902	1.6270
**Distance (m) at absolute HSR per minute**	9.5200	3.2662	9.6428	3.4331	0.0158	2.5608
**Maximal speed (km/h)**	30.4345	1.7251	29.8775	1.7868	-0.4775	1.3424
**Distance (m) covered in absolute sprinting per minute**	0.6997	0.4567	0.8656	0.5704	0.1667	0.6157
**Composite index (AU)**	15366.0867	6295.4249	12714.7035	5391.9674	-2765.7505	6506.5696
**Composite index (AU) per minute**	244.2295	79.9088	247.8683	72.4906	-1.1772	64.2770

HSR = high-speed running. Absolute threshold values for Sprint and HSR were > 24 km/h and > 21 km/h, respectively. Relative threshold values referred to > 85% of the player’s maximum speed based on the WIMU profile.

### Pre- and post-injury differences

To assess whether the differences between pre- and post-injury performance metrics were statistically significant, we first evaluated the normality of each metric’s difference distribution using the Shapiro-Wilk test (α = 0.05). For metrics with a normal difference distribution, a paired t-test was applied, while the non-parametric Wilcoxon signed-rank test was used for metrics that did not meet the normality assumption. Table 2 provides an overview of the statistical test results for each metric, indicating significant differences between pre- and post-injury averages.

**TABLE 2:** Statistical test results for pre- and post-injury differences

Metric	Shapiro-Wilk statistic	Shapiro-Wilk P-value	Test used	Test statistic	P-value	Significant difference
**Time (min)**	0.9923	0.7942	T-test	6.3653	< 0.0001	**Yes**
**Distance (m/min)**	0.9882	0.5892	T-test	-2.0006	0.0485	**Yes**
**Acceleration counts per minute**	0.9918	0.8505	T-test	-0.2342	0.8154	No
**Deceleration counts per minute**	0.9913	0.8191	T-test	-0.0186	0.9852	No
**Maximal acceleration (m/s^2^)**	0.9834	0.3014	T-test	2.6567	0.0093	**Yes**
**Maximal deceleration (m/s^2^)**	0.9891	0.6583	T-test	-2.5768	0.0116	**Yes**
**Distance (m) accelerating at HI per minute**	0.9440	0.0007	Wilcoxon signed-rank test	2059.0000	0.8930	No
**Distance (m) decelerating at HI per minute**	0.9897	0.7058	T-test	-0.1761	0.8606	No
**Count of absolute sprinting actions per minute**	0.9791	0.1509	T-test	0.5795	0.5637	No
**Count of relative HSR actions per minute**	0.9419	0.0005	Wilcoxon signed-rank test	1674.0000	0.0972	No
**Count of absolute HSR actions per minute**	0.9904	0.7522	T-test	-0.2539	0.8002	No
**Count of relative sprinting actions per minute**	0.9154	0.0000	Wilcoxon signed-rank test	466.0000	0.0104	**Yes**
**Distance (m) at relative HSR per minute**	0.9747	0.0737	T-test	0.5291	0.5981	No
**Distance (m) at absolute HSR per minute**	0.9877	0.5552	T-test	-0.0590	0.9531	No
**Maximal speed (km/h)**	0.9916	0.8391	T-test	3.3931	0.0010	**Yes**
**Distance (m) covered in absolute sprinting per minute**	0.9442	0.0007	Wilcoxon signed-rank test	1390.0000	0.0054	**Yes**
**Composite index (AU)**	0.9878	0.5655	T-test	4.0549	0.0001	**Yes**
**Composite index (AU) per minute**	0.9924	0.8880	T-test	0.1747	0.8617	No

HSR = high-speed running. Absolute threshold values for Sprint and HSR were > 24 km/h and > 21 km/h, respectively. Relative threshold values referred to > 85% of the player’s maximum speed based on the WIMU profile.

Metrics showing significant pre- and post-injury differences (P < 0.05) included time played, total distance covered, maximal acceleration, maximal deceleration, number of relative sprint actions, maximal speed, distance covered sprinting, and the composite index. These results suggest that these parameters were meaningfully impacted by injury, warranting further analysis in relation to absence time.

### Correlation analysis with absence time

To explore the relationship between absence time and performance changes, variables that showed statistically significant differences between pre- and post-injury averages were included in a correlation analysis. Table 3 presents the correlation coefficients between absence time and each significant performance metric difference.

**TABLE 3:** Pearson correlation coefficients between recovery time and significant pre-post performance metric differences

Metric difference	Correlation with absence time
Difference in maximal speed	-0.355
Difference in time played	-0.328
Difference in maximal acceleration	-0.303
Difference in composite index	-0.218
Difference in distance covered sprinting	0.205
Difference in maximal deceleration	0.185
Difference in number of relative sprint actions	-0.149
Difference in distance covered	0.078

Absolute threshold values for Sprint was > 24 km/h. Relative threshold values referred to > 85% of the player’s maximum speed based on the WIMU profile.

The strongest correlation was observed for the difference in maximal speed (r = -0.355), indicating that longer recovery times were associated with a more pronounced reduction in maximum speed. This was followed by the difference in time played (r = -0.328) and the difference in maximal acceleration (r = -0.303), suggesting that extended recovery durations are linked to decreases in both playing time and maximal acceleration. Additional negative correlations were found for the difference in composite index (r = -0.218) and the difference in the number of sprints with relative threshold performed (r = -0.149), pointing to declines in composite performance and relative sprint counts as absence time increases. In contrast, positive although weaker correlations were found for the difference in the distance sprinting (r = 0.205) and the difference in maximal deceleration (r = 0.185). Overall, these findings suggest that extended recovery times tend to correlate with reductions in high-intensity performance metrics, particularly in maximum speed and acceleration, highlighting areas most impacted by prolonged absences.

### Machine learning analysis

To further understand and model the relationships between those performance metrics that seem to worsen as the recovery period extends, a machine learning analysis was conducted. While statistical analysis highlighted significant differences between pre- and post-injury performance metrics, machine learning allows us to identify and quantify the features most associated with the length of recovery time. Two distinct regression models were employed to assess the relationships between the change in performance metrics and recovery time: MLR and RFR. The MSE and R^2^ scores for both models on the training and test sets are presented below:

– **MLR MSE**: Training set = 689.057; Test set = 365.1421
– **RFR MSE**: Training set = 514.169; Test set = 312.355
– **MLR R^2^**: Training set = 0.1163; Test set = 0.348
– **RFR R^2^**: Training set = 0.341; Test set = 0.442

The RFR model demonstrated a lower MSE and higher R^2^ score compared to the MLR model, indicating better performance in capturing the relationships between performance change and recovery time. This improvement in RFR’s performance is likely due to its ability to model complex, non-linear relationships among the features, whereas MLR assumes linearity and independence among predictors. In this case, the independent variables may not be entirely independent, negatively affecting the MLR’s accuracy.

**FIG. 1:**
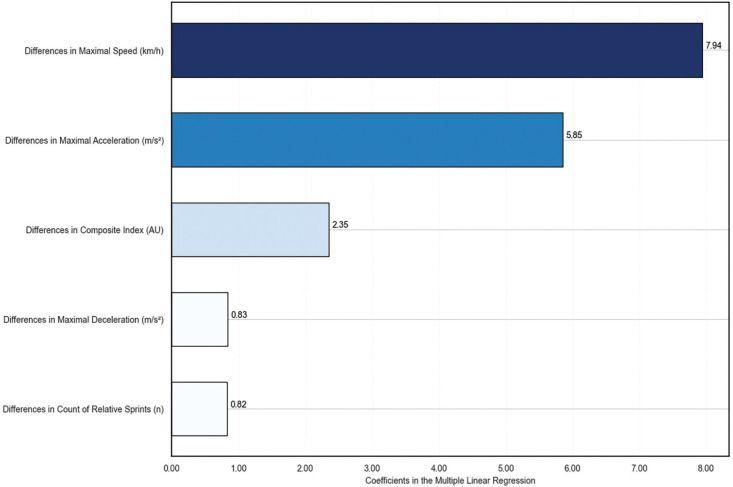
Multiple linear regression coefficients, indicating the relative impact of each parameter on the duration of absence.

To interpret the models, Figure 1 presents a visual representation of the MLR coefficients for each variable, sorted by absolute value. This helps to identify which variables are most strongly correlated with recovery time. The MLR model estimates the absence time using the following linear equation:

Absence time [days] = β0 + β1 · Differences in Maximal Acceleration + β2 · Differences in Maximal Deceleration + β3 · Differences in Number of Relative Sprint Actions + β4 · Differences in Maximal Speed + β5 · Differences in Composite Index

**FIG. 2:**
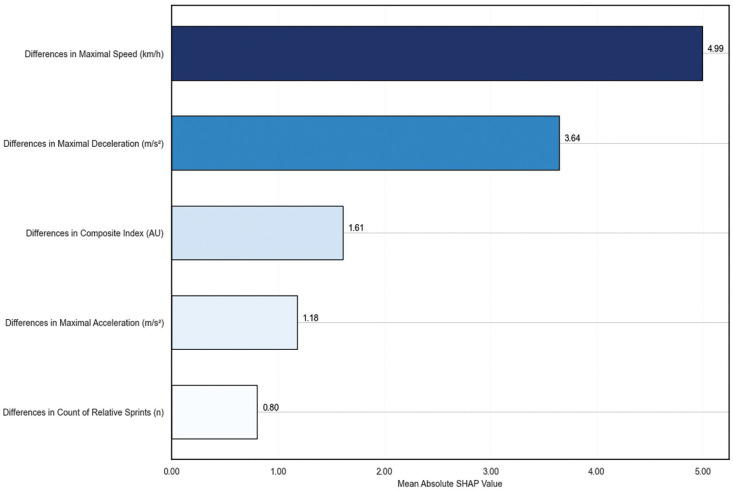
Mean absolute SHAP values from the random forest regression. Higher SHAP values indicate features that contribute more to the model’s predictions of absence duration.

**FIG. 3:**
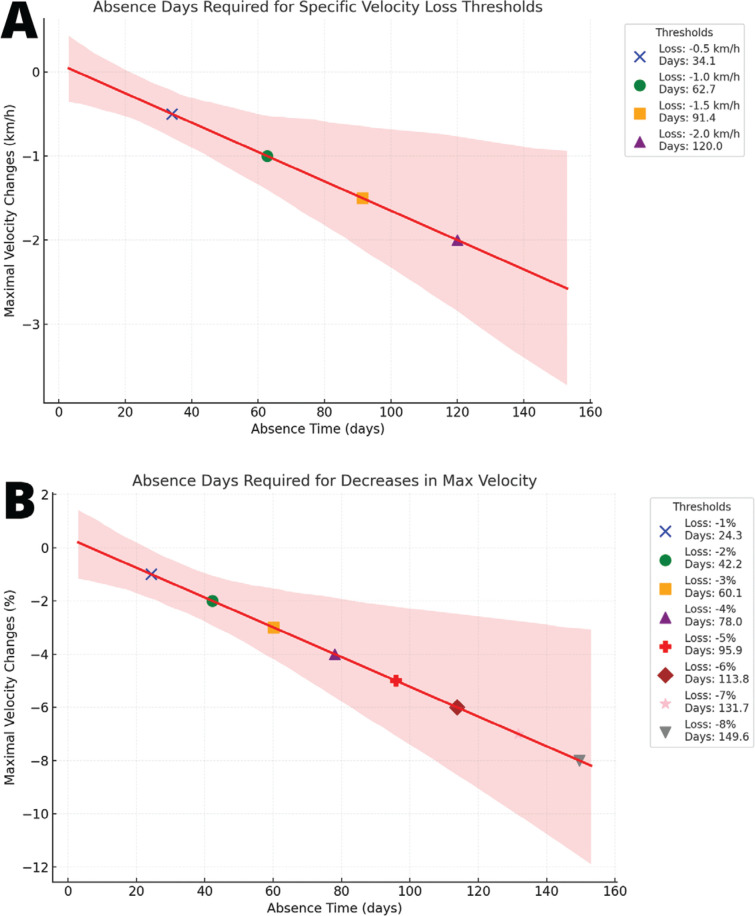
Linear regression models illustrating the effects of absence time on maximal speed changes, with 95% confidence intervals. Plot A shows changes in absolute values (km/h), while plot B represents changes in percentages of pre-injury values.

In this model, the β coefficients represent the estimated change in absence time for each unit change in the respective independent variable, assuming all other variables remain constant. β0, β1, β2, β3, β4 and β5 showed a value of 33.22, -5.85, -0.83, 0.82, -7.94 and 2.35, respectively. For the RFR model, SHAP values were used to determine the contribution of each feature to the prediction of recovery time, as shown in Figure 2. Both models consistently identified the difference in maximal speed as a key factor related to recovery time, suggesting that this metric may be particularly affected by longer recovery periods. Linear regression with 95% confidence interval of maximal speed (i.e., as the variable with better association with absence time in machine learning models) was plotted both in relative (%) and absolute (km/h) changes in Figure 3.

## DISCUSSION

### The effect of absence time on loss of performance

This study aimed to explain which differences in external load metrics are more strongly associated with absence time in elite soccer players after muscle injuries. Our results, derived from machine learning algorithms, suggest that absence time is associated with the loss of maximal speed, with longer absences leading to greater performance losses in this metric during matches. The results of the present study could be important to better understand what the consequences of a muscle injury are depending on its absence time. Practitioners can expect a larger decrease in maximal speed and deceleration/acceleration outcomes when the recovery process is longer, thus adapting their reconditioning strategies to perform better in subsequent matches. Given this fact, two players with a hamstring injury but differing prognoses should follow distinct return-to-play pathways. The player with a longer recovery period is likely to experience greater losses in maximal speed and acceleration/deceleration abilities during matches. Consequently, return-to-play criteria for this player should prioritize specific tests to ensure recovery of pre-injury levels of maximal speed and acceleration/deceleration [51]. These assessments should play a more prominent role in their rehabilitation process compared to a player with a shorter recovery timeline [51–53]. By adopting this approach, practitioners can optimize the rehabilitation process, facilitating quicker return-to-performance by ensuring the athlete regains pre-injury performance levels as efficiently as possible.

The loss of performance after muscle injuries has been previously reported. In line with our results, two studies showed reductions in maximal speed [26, 28]. Nonetheless, this information was not linked to the implicit variability in absence time related to muscle injuries. Our results clearly showed that maximal speed is the variable that is more closely linked to longer absence times, while maximal acceleration and deceleration can also be influenced by absence time. Interestingly, the difference in the number of sprints at the relative threshold (i.e., 85% of the player’s maximum speed) was the variable that showed the weakest relationship with absence time in our two machine learning models. This means that longer periods of recovery are not necessarily linked to greater loss of performance in this metric, so practitioners can expect similar decreases in the number of sprints performed regardless of the absence time. Nonetheless, practitioners should assess whether an athlete’s maximal speed and acceleration/deceleration capacity have returned to pre-injury levels. The composite index shows that the overall performance of the player is affected by muscle injury, but this decrease could be partly explained by the shorter duration of time played, as shown in our pre-post injury difference analyses. However, it seems that the longer the absence time, the greater the loss of overall performance (i.e., composite index), which is important to note.

### Loss of performance during matches: implications and solutions

Decreases in maximal speed, maximal acceleration/deceleration, overall performance (i.e., composite index) and number of sprints were observed in our analyses. Notably, most of the recorded injuries (52 out of 110 injuries) affected the hamstrings. Maximal speed is the variable that demonstrated the largest decrease with longer absence times, which is closely related to the activity and function of the hamstrings [54–56]. Therefore, as previously reported, practitioners should check whether maximal sprinting velocity has been recovered in analytical tests (i.e., isolated linear sprints) [57, 58]. Nonetheless, previous research has established that previously injured players showed decreases in the acceleration phase (i.e., ability linked to maximal horizontal force production) rather than in the maximal speed phase [59, 60]. However, our results explicitly demonstrated that maximal acceleration ability is affected during matches, and that absence time largely explains the loss of performance in this metric (i.e., longer recovery periods lead to larger decreases in maximal acceleration). As shown in Figure 3, linear regression models clearly illustrate the downward trend in maximal speed differences as absence time increases. However, it is notable that variability also increases with longer absence periods, making changes in maximal speed more unpredictable with extended recovery times. Therefore, coaches should be particularly aware of this variability, especially in cases of prolonged absences due to injury, which often involve tendon tissue and present greater challenges for prognosis [52, 53]. Although the literature in this field is still scarce, lower acceleration could lead to less achievement of maximal speed during matches, since sprints in soccer are mainly performed for shorter distances (i.e., 2 to 4 s or 10 to 30 m) than those covered to assess mechanical sprinting properties (i.e., 40 m) [59, 60]. Therefore, given the short distances where sprints occur in soccer and given the reduced time for performing them, a loss of maximal acceleration could be linked to a reduced maximal speed outcome during matches (i.e., there is no time and space for achieving maximal speed). Given this association, it is crucial for practitioners to assess mechanical properties of sprinting prior to return to play [61]. However, it is also important to note that most of the sprints in soccer are not in a linear pattern (i.e., approximately 85% of maximum velocity manoeuvres involve curvilinear sprints) [62, 63], with torso rotation (62% of sprints) [62] and ending with an action such as duelling with an opponent or involvement with the ball (50% of the sprints) [62]. Therefore, it is important to achieve peaks of maximal speed and accelerations in integrated soccer tasks such as transition games [64], one-on-one transition tasks [65] or small-sided games [66, 67]. Regarding assessment of specific sprinting patterns in soccer, it is important to assess curvilinear sprinting tests [68] and repeated sprinting ability, recreating the specific demands of the game [69]. In addition, Global Positioning System (i.e., external load) metrics should be checked during late stages of the rehabilitation process to ensure that preinjury maximal speed and acceleration/deceleration output has been reached [70, 71].

The fact that maximal deceleration has been identified as the second most modifiable variable depending on absence time in our RFR model is highly relevant. This could be attributed to the longer absence time in those muscle injuries that mostly involve the tendon [18, 52, 53]. It is well known that high-intensity braking actions are highly dependent on the tendon capacity [72, 73], which is linked to the eccentric muscle contraction capacity [74, 75]. Based on our machine learning models and in these associations, it is crucial to check eccentric strength ability, as well as integrating it into high-demanding braking on-field activities before returning to play after muscle injuries [72]. The fact that maximal deceleration ability is more affected by longer rehabilitation processes could be associated with maladaptation in tendon capacity due to the lack of mechanical stimulus [76, 77]. Therefore, this is especially relevant in those injuries affecting more tendon tissue (associated with longer absence times). The loss of maximal braking ability is also linked to increased knee joint mechanical loading during the final foot contact of changes of direction [78]. Therefore, longer muscle injuries can potentially increase the risk of knee injuries if the loss of maximal deceleration during matches is produced due to an incapacity in reaching high-intensity deceleration values, especially after hamstring injuries [79]. Consequently, it is crucial to check maximal deceleration ability before the return to play to avoid severe injuries in other tissues such as the anterior cruciate ligament [79]. A potential solution for this issue is to introduce early eccentric exercises, which have been demonstrated to be safe during rehabilitation of muscle injuries [80]. In addition, flywheel resistance training during rehabilitation and especially braking in the last third of the movement (i.e., lengthening position) could be of interest in longer rehabilitation periods produced by tendon tissue involvement [81, 82].

While maximum speed, acceleration, and deceleration capacity are significantly influenced by absence time, our machine learning models show that the number of sprints performed is not dependent on the recovery time. This finding was surprising given that both repeated sprint ability [83] and maximal eccentric strength [84] have not been found to be impaired after injury. Therefore, the cause of the lower number of sprinting actions is not clear in our opinion. Anyway, this outcome should be checked regardless of the absence time, since shorter recovery periods can produce similar decreases in the number of sprinting actions performed. In this regard, Whiteley et al. [29] proposed that there may be additional return to sport criteria for some players in terms of high-speed running or sprinting, which aligns with the results of our study. Moreover, the shared decision-making model of return to sport highlighted the “ability to perform” [85], which is not being met based on the findings of the present study. Consequently, practitioners should consider not only clinical outcomes for avoiding reinjuries but also performance-based metrics such as the ability to perform several sprints, independently of the absence time. This aligns with the return-to-performance approach, which emphasizes not just medical clearance but the full restoration of key physical capacities essential for optimal soccer performance [31, 32].

### Clinical recommendations

This study emphasized the importance of assessing the following outcomes as criteria for return to play, especially as the length of absence increases.

1. Ability to reach similar pre-injury maximal speed
2. Ability to perform similar pre-injury maximal decelerations
3. Ability to perform similar pre-injury maximal accelerations.
  In addition, regardless of the absence time, it is always important to check:
4. Ability to perform similar pre-injury sprints during matches or in integrated soccer-specific tasks.

## CONCLUSIONS

The findings of the present study suggest that prolonged recovery times after muscle injuries are associated with reductions in maximum speed and acceleration/deceleration capacity in elite soccer players. However, the number of sprinting actions did not show relationships with absence time, suggesting that this outcome should be assessed regardless of the recovery time. By focusing on these high-impact performance metrics during rehabilitation and taking absence time as an important factor for individualizing return to play criteria and rehabilitation progression, practitioners may be able to develop targeted interventions that expedite recovery and mitigate performance losses after injury. These findings can contribute to the design of the return-to-performance phase, helping to bridge the gap between return-to-play and full restoration of pre-injury performance levels.

## Data Availability

Data for the preparation of this study could be provided after reasonable request to the authors.

## References

[1] Sanchez-Trigo H, Molina-Martínez E, Grimaldi-Puyana M, et al. Effects of lifestyle behaviours and depressed mood on sleep quality in young adults. A machine learning approach. Psychol Health. 2024; 39(1):128–143. 35475409 10.1080/08870446.2022.2067331

[2] Edouard P, Verhagen E, Navarro L. Machine learning analyses can be of interest to estimate the risk of injury in sports injury and rehabilitation. Ann Phys Rehabil Med. 2022; 65(4):101431. 32871283 10.1016/j.rehab.2020.07.012

[3] Oliver JL, Ayala F, De Ste Croix MBA, et al. Using machine learning to improve our understanding of injury risk and prediction in elite male youth football players. J Sci Med Sport. 2020; 23(11):1044–1048. 32482610 10.1016/j.jsams.2020.04.021

[4] Lemon SC, Roy J, Clark MA, et al. Classification and regression tree analysis in public health: Methodological review and comparison with logistic regression. Annals of Behavioral Medicine. 2003; 26(3):172–181. 14644693 10.1207/S15324796ABM2603_02

[5] Liu Y, Chen P-HC, Krause J, et al. How to Read Articles That Use Machine Learning. JAMA. 2019; 322(18):1806. 31714992 10.1001/jama.2019.16489

[6] Bunker RP, Thabtah F. A machine learning framework for sport result prediction. Applied Computing and Informatics. 2019; 15(1):27–33.

[7] Helm JM, Swiergosz AM, Haeberle HS, et al. Machine learning and artificial intelligence: definitions, applications, and future directions. Curr Rev Musculoskelet Med. 2020; 13:69–76. 31983042 10.1007/s12178-020-09600-8 PMC7083992

[8] Leckey C, van Dyk N, Doherty C, et al. Machine learning approaches to injury risk prediction in sport: a scoping review with evidence synthesis. Br J Sports Med. 2024; bjsports-2024-108576. 10.1136/bjsports-2024-108576 PMC12013557 39613453

[9] Collins GS, Moons KGM. Reporting of artificial intelligence prediction models. The Lancet. 2019; 393(10181):1577–1579. 10.1016/S0140-6736(19)30037-6 31007185

[10] Aksu G, Keceoglu CR. Comparison of Results Obtained from Logistic Regression, CHAID Analysis and Decision Tree Methods. Eurasian Journal of Educational Research. 2019; 19(84):1–20.

[11] Rommers N, Rössler R, Verhagen E, et al. A Machine Learning Approach to Assess Injury Risk in Elite Youth Football Players. Med Sci Sports Exerc. 2020; 52(8):1745–1751. 32079917 10.1249/MSS.0000000000002305

[12] Pillitteri G, Petrigna L, Ficarra S, et al. Relationship between external and internal load indicators and injury using machine learning in professional soccer: a systematic review and meta-analysis. Research in Sports Medicine. 2023; 1–37. 38146925 10.1080/15438627.2023.2297190

[13] Warren P, Gabbe BJ, Schneider-Kolsky M, et al. Clinical predictors of time to return to competition and of recurrence following hamstring strain in elite Australian footballers. Br J Sports Med. 2010; 44(6):415–419. 18653619 10.1136/bjsm.2008.048181

[14] McAuley S, Dobbin N, Morgan C, et al. Predictors of time to return to play and re-injury following hamstring injury with and without intramuscular tendon involvement in adult professional footballers: A retrospective cohort study. J Sci Med Sport. 2022; 25(3):216–221. 34740516 10.1016/j.jsams.2021.10.005

[15] Pollock N, Patel A, Chakraverty J, et al. Time to return to full training is delayed and recurrence rate is higher in intratendinous (‘c’) acute hamstring injury in elite track and field athletes: clinical application of the British Athletics Muscle Injury Classification. Br J Sports Med. 2016; 50(5):305–310. 26888072 10.1136/bjsports-2015-094657

[16] Pollock N, James SLJ, Lee JC, et al. British athletics muscle injury classification: a new grading system. Br J Sports Med. 2014; 48(18):1347–1351. 25031367 10.1136/bjsports-2013-093302

[17] Macdonald B, McAleer S, Kelly S, et al. Hamstring rehabilitation in elite track and field athletes: applying the British Athletics Muscle Injury Classification in clinical practice. Br J Sports Med. 2019; 53(23):1464–1473. 31300391 10.1136/bjsports-2017-098971

[18] Shamji R, James SLJ, Botchu R, et al. Association of the British Athletic Muscle Injury Classification and anatomic location with return to full training and reinjury following hamstring injury in elite football. BMJ Open Sport Exerc Med. 2021; 7(2):e001010. 10.1136/bmjsem-2020-001010 PMC8112435 34040793

[19] Ekstrand J, Spreco A, Bengtsson H, et al. Injury rates decreased in men’s professional football: an 18-year prospective cohort study of almost 12 000 injuries sustained during 1.8 million hours of play. Br J Sports Med. 2021; 55(19):1084–1092. 33547038 10.1136/bjsports-2020-103159 PMC8458074

[20] Ekstrand J, Hagglund M, Walden M. Injury incidence and injury patterns in professional football: the UEFA injury study. Br J Sports Med. 2011; 45(7):553–558. 19553225 10.1136/bjsm.2009.060582

[21] Ekstrand J, Bengtsson H, Waldén M, et al. Hamstring injury rates have increased during recent seasons and now constitute 24% of all injuries in men’s professional football: the UEFA Elite Club Injury Study from 2001/02 to 2021/22. Br J Sports Med. 2023; 57(5):292–298. 10.1136/bjsports-2021-105407 PMC9985757 36588400

[22] Ekstrand J, Hägglund M, Waldén M. Epidemiology of Muscle Injuries in Professional Football (Soccer). Am J Sports Med. 2011; 39(6):1226–1232. 21335353 10.1177/0363546510395879

[23] López-Valenciano A, Ruiz-Pérez I, Garcia-Gómez A, et al. Epidemiology of injuries in professional football: a systematic review and meta-analysis. Br J Sports Med. 2020; 54(12):711–718. 31171515 10.1136/bjsports-2018-099577 PMC9929604

[24] Diemer WM, Winters M, Tol JL, et al. Incidence of Acute Hamstring Injuries in Soccer: A Systematic Review of 13 Studies Involving More Than 3800 Athletes With 2 Million Sport Exposure Hours. Journal of Orthopaedic & Sports Physical Therapy. 2021; 51(1):27–36. 33306929 10.2519/jospt.2021.9305

[25] Raya-González J, Pulido JJ, Beato M, et al. Analysis of the Effect of Injuries on Match Performance Variables in Professional Soccer Players: A Retrospective, Experimental Longitudinal Design. Sports Med Open. 2022; 8(1):31. 35239035 10.1186/s40798-022-00427-w PMC8894514

[26] Morgans R, Rhodes D, Bezuglov E, et al. The impact of injury on match running performance following the return to competitive match-play over two consecutive seasons in elite European soccer players. Journal of physical education and sport. 2023; 23(5):1142–1149.

[27] Valera-Garrido F, Jiménez-Rubio S, Minaya-Muñoz F, et al. Ultrasound-Guided Percutaneous Needle Electrolysis and Rehab and Reconditioning Program for Rectus Femoris Muscle Injuries: A Cohort Study with Professional Soccer Players and a 20-Week Follow-Up. Applied Sciences. 2020; 10(21):7912.

[28] Whiteley R, Gregson W, Bahr R, et al. High-speed running during match-play before and after return from hamstring injury in professional footballers. Scand J Med Sci Sports. 2022; 32(10):1502–1509. 35934809 10.1111/sms.14219

[29] Whiteley R, Massey A, Gabbett T, et al. Match High-Speed Running Distances Are Often Suppressed After Return From Hamstring Strain Injury in Professional Footballers. Sports Health: A Multidisciplinary Approach. 2021; 13(3):290–295. 10.1177/1941738120964456 PMC8079800 33151808

[30] Hills SP, Barwood MJ, Radcliffe JN, et al. Profiling the Responses of Soccer Substitutes: A Review of Current Literature. Sports Medicine. 2018; 48(10):2255–2269. 30051171 10.1007/s40279-018-0962-9

[31] Mitchell A, Gimpel M. A Returnto-Performance Pathway for Professional Soccer: A Criteria-based Approach to Return Injured Professional Players Back to Performance. JOSPT Open. 2024; 2(3):166–178.

[32] Dixon B, Alexander J, Harper D. ‘Post-rehabilitation phase’ in professional football: are we optimising player support after return to play? Br J Sports Med. 2025; bjsports-2024-109458. 10.1136/bjsports-2024-109458 39880605

[33] Perez-Arroniz M, Calleja-González J, Zabala-Lili J, et al. The soccer goalkeeper profile: bibliographic review. Phys Sportsmed. 2023; 51(3):193–202. 35157536 10.1080/00913847.2022.2040889

[34] Bush M, Barnes C, Archer DT, et al. Evolution of match performance parameters for various playing positions in the English Premier League. Hum Mov Sci. 2015; 39:1–11. 25461429 10.1016/j.humov.2014.10.003

[35] Barrera J, Sarmento H, Clemente FM, et al. The Effect of Contextual Variables on Match Performance across Different Playing Positions in Professional Portuguese Soccer Players. Int J Environ Res Public Health. 2021; 18(10):5175. 34068150 10.3390/ijerph18105175 PMC8152996

[36] Guerrero-Calderón B, Alfonso Morcillo J, Chena M, et al. Comparison of training and match load between metabolic and running speed metrics of professional Spanish soccer players by playing position. Biol Sport. 2022; 39(4):933–941. 36247950 10.5114/biolsport.2022.110884 PMC9536366

[37] Oliva-Lozano JM, Fortes V, López-Del Campo R, et al. When and How do Professional Soccer Players Experience Maximal Intensity Sprints in Laliga? Science and Medicine in Football. 2022; doi: 10.1080/24733938.2022.2100462. 35803616

[38] Reverte-Pagola G, Pecci J, del Ojo-López JJ, et al. Analyzing the impact of non-participation in the FIFA World Cup Qatar 2022 on LaLiga players’ physical performance. Front Sports Act Living. 2024; 6. 10.3389/fspor.2024.1385267 PMC11026628 38645722

[39] Errekagorri I, Fernandez-Navarro J, López-Del Campo R, et al. An eight-season analysis of the teams’ performance in the Spanish LaLiga according to the final league ranking. PLoS One. 2024; 19(2):e0299242. 38416760 10.1371/journal.pone.0299242 PMC10901331

[40] Brito de Souza D, López-Del Campo R, Resta R, et al. Running Patterns in LaLiga Before and After Suspension of the Competition Due to COVID-19. Front Physiol. 2021; 12. 10.3389/fphys.2021.666593 PMC8107438 33981253

[41] Oliva-Lozano JM, Cefis M, Fortes V, et al. Summarizing physical performance in professional soccer: development of a new composite index. Sci Rep. 2024; 14(1):14453. 38914672 10.1038/s41598-024-65581-5 PMC11196579

[42] Eberly LE. Multiple Linear Regression. In: Ambrosius WT, editor. Topics in Biostatistics [Internet]. Totowa, NJ: Humana Press; 2007. p. 165–187. Available from: 10.1007/978-1-59745-530-5_9.

[43] Nohara Y, Matsumoto K, Soejima H, et al. Explanation of machine learning models using shapley additive explanation and application for real data in hospital. Comput Methods Programs Biomed. 2022; 214:106584. 34942412 10.1016/j.cmpb.2021.106584

[44] Vittinghoff E, Glidden D V., Shiboski SC, et al. Regression Methods in Biostatistics. Boston, MA: Springer US; 2012.

[45] Hastie T, Tibshirani R, Friedman J. The Elements of Statistical Learning. New York, NY: Springer New York; 2009.

[46] Smith PF, Ganesh S, Liu P. A comparison of random forest regression and multiple linear regression for prediction in neuroscience. J Neurosci Methods. 2013; 220(1):85–91. 24012917 10.1016/j.jneumeth.2013.08.024

[47] Yuchi W, Gombojav E, Boldbaatar B, et al. Evaluation of random forest regression and multiple linear regression for predicting indoor fine particulate matter concentrations in a highly polluted city. Environmental pollution. 2019; 245:746–753. 30500754 10.1016/j.envpol.2018.11.034

[48] Lundberg S. A unified approach to interpreting model predictions. arXiv preprint arXiv:170507874. 2017.

[49] Kim Y, Kim Y. Explainable heat-related mortality with random forest and SHapley Additive exPlanations (SHAP) models. Sustain Cities Soc. 2022; 79:103677.

[50] García MV, Aznarte JL. Shapley additive explanations for NO_2_ forecasting. Ecol Inform. 2020; 56:101039.

[51] Perna P, Kerin F, Greig N, et al. Return-to-play criteria following a hamstring injury in professional football: a scoping review. Research in Sports Medicine. 2024; 1–20. 10.1080/15438627.2024.2439274 39666593

[52] Kerin F, O’Flanagan S, Coyle J, et al. Intramuscular Tendon Injuries of the Hamstring Muscles: A More Severe Variant? A Narrative Review. Sports Med Open. 2023; 9(1):75. 37578668 10.1186/s40798-023-00621-4 PMC10425319

[53] Beattie CE, Barnett RJ, Williams J, et al. Are return-to-play times longer in lower-limb muscle injuries involving the intramuscular tendon? A systematic review. J Sci Med Sport. 2023; 26(11):599–609. 37884432 10.1016/j.jsams.2023.10.002

[54] Huygaerts S, Cos F, Cohen DD, et al. Mechanisms of Hamstring Strain Injury: Interactions between Fatigue, Muscle Activation and Function. Sports. 2020; 8(5):65. 32443515 10.3390/sports8050065 PMC7281534

[55] Garcia AG, Andrade R, Afonso J, et al. Hamstrings injuries in football. J Orthop. 2022; 31:72–77. 35464813 10.1016/j.jor.2022.04.003 PMC9026901

[56] Whiteley R, van Dyk N, Wangensteen A, et al. Clinical implications from daily physiotherapy examination of 131 acute hamstring injuries and their association with running speed and rehabilitation progression. Br J Sports Med. 2018; 52(5):303–310. 29084725 10.1136/bjsports-2017-097616

[57] Mendiguchia J, Brughelli M. A return-tosport algorithm for acute hamstring injuries. Physical Therapy in Sport. 2011; 12(1):2–14. 21256444 10.1016/j.ptsp.2010.07.003

[58] Mendiguchia J, Martinez-Ruiz E, Edouard P, et al. A Multifactorial, Criteria-based Progressive Algorithm for Hamstring Injury Treatment. Med Sci Sports Exerc. 2017; 49(7):1482–1492. 28277402 10.1249/MSS.0000000000001241

[59] Mendiguchia J, Samozino P, Martinez-Ruiz E, et al. Progression of Mechanical Properties during On-field Sprint Running after Returning to Sports from a Hamstring Muscle Injury in Soccer Players. Int J Sports Med. 2014; 35(08):690–695. 24424959 10.1055/s-0033-1363192

[60] Mendiguchia J, Edouard P, Samozino P, et al. Field monitoring of sprinting power–force–velocity profile before, during and after hamstring injury: two case reports. J Sports Sci. 2016; 34(6):535–541. 26648237 10.1080/02640414.2015.1122207

[61] Mendiguchia J, Garrues MA, Schilders E, et al. Anterior pelvic tilt increases hamstring strain and is a key factor to target for injury prevention and rehabilitation. Knee Surgery, Sports Traumatology, Arthroscopy. 2024; 32(3):573–582. 10.1002/ksa.12045 38391038

[62] Caldbeck P, Dos’Santos T. A classification of specific movement skills and patterns during sprinting in English Premier League soccer. PLoS One. 2022; 17(11):e0277326. 36367861 10.1371/journal.pone.0277326 PMC9651586

[63] Solleiro-Duran D, Cidre-Fuentes P, Rey E, et al. Effects of linear versus curvilinear sprint training on multidirectional speed in young soccer players: a randomized parallel-group trial. Biol Sport. 2025; doi: 10.5114/biolsport.2025.139084. PMC11694198 39758166

[64] Asian-Clemente JA, Rabano-Muñoz A, Suarez-Arrones L, et al. Different pitch configurations constrain the external and internal loads of young professional soccer players during transition games. Biol Sport. 2023; 40(4):1047–1055. 37867736 10.5114/biolsport.2023.124848 PMC10588570

[65] Asian-Clemente J, Rabano-Munoz A, Requena B, et al. Effects of Bout Duration on Load, Sprint, and Jump Ability During a One-on-one Transition Task. Int J Sports Med. 2023; 44(08):568–575. 36809786 10.1055/a-2040-2578

[66] Skala F, Zemková E. Neuromuscular and perceptual-cognitive response to 4 v 4 small-sided game in youth soccer players. Front Physiol. 2023; 14. 10.3389/fphys.2023.1260096 PMC10665484 38028786

[67] Beenham M, Barron DJ, Fry J, et al. A Comparison of GPS Workload Demands in Match Play and Small-Sided Games by the Positional Role in Youth Soccer. J Hum Kinet. 2017; 57(1):129–137. 28713465 10.1515/hukin-2017-0054 PMC5504585

[68] Fílter A, Olivares J, Santalla A, et al. New curve sprint test for soccer players: Reliability and relationship with linear sprint. J Sports Sci. 2020; 38(11–12):1320–1325. 31607228 10.1080/02640414.2019.1677391

[69] Schimpchen J, Skorski S, Nopp S, et al. Are “classical” tests of repeated-sprint ability in football externally valid? A new approach to determine in-game sprinting behaviour in elite football players. J Sports Sci. 2016; 34(6):519–526. 26580089 10.1080/02640414.2015.1112023

[70] Buckthorpe M, Della Villa F, Della Villa S, et al. On-field Rehabilitation Part 2: A 5-Stage Program for the Soccer Player Focused on Linear Movements, Multidirectional Movements, Soccer-Specific Skills, Soccer-Specific Movements, and Modified Practice. Journal of Orthopaedic & Sports Physical Therapy. 2019; 49(8):570–575. 31291556 10.2519/jospt.2019.8952

[71] Armitage M, McErlain-Naylor SA, Devereux G, et al. On-field rehabilitation in football: Current knowledge, applications and future directions. Front Sports Act Living. 2022; 4. 10.3389/fspor.2022.970152 PMC9760760 36544545

[72] McBurnie AJ, Harper DJ, Jones PA, et al. Deceleration Training in Team Sports: Another Potential ‘Vaccine’ for Sports-Related Injury? Sports Medicine. 2022; 52(1):1–12. 10.1007/s40279-021-01583-x PMC8761154 34716561

[73] Burton I. Interventions for prevention and in-season management of patellar tendinopathy in athletes: A scoping review. Physical Therapy in Sport. 2022; 55:80–89. 35286941 10.1016/j.ptsp.2022.03.002

[74] Jones P, Thomas C, Dos’Santos T, et al. The Role of Eccentric Strength in 180° Turns in Female Soccer Players. Sports. 2017; 5(2):42. 29910402 10.3390/sports5020042 PMC5968983

[75] de Hoyo M, de la Torre A, Pradas F, et al. Effects of Eccentric Overload Bout on Change of Direction and Performance in Soccer Players. Int J Sports Med. 2014; 36(04):308–314. 25525954 10.1055/s-0034-1395521

[76] Maganaris CN, Chatzistergos P, Reeves ND, et al. Quantification of Internal Stress-Strain Fields in Human Tendon: Unraveling the Mechanisms that Underlie Regional Tendon Adaptations and Mal-Adaptations to Mechanical Loading and the Effectiveness of Therapeutic Eccentric Exercise. Front Physiol. 2017; 8. 28293194 10.3389/fphys.2017.00091 PMC5328946

[77] Logerstedt DS, Ebert JR, MacLeod TD, et al. Effects of and Response to Mechanical Loading on the Knee. Sports Medicine. 2022; 52(2):201–235. 34669175 10.1007/s40279-021-01579-7

[78] Dos’Santos T, Thomas C, Comfort P, et al. The Effect of Angle and Velocity on Change of Direction Biomechanics: An Angle-Velocity Trade-Off. Sports Medicine. 2018; 48(10):2235–2253. 30094799 10.1007/s40279-018-0968-3 PMC6132493

[79] Opar DA, Serpell BG. Is There a Potential Relationship Between Prior Hamstring Strain Injury and Increased Risk for Future Anterior Cruciate Ligament Injury? Arch Phys Med Rehabil. 2014; 95(2):401–405. 24121082 10.1016/j.apmr.2013.07.028

[80] Vermeulen R, Whiteley R, van der Made AD, et al. Early versus delayed lengthening exercises for acute hamstring injury in male athletes: a randomised controlled clinical trial. Br J Sports Med. 2022; 56(14):792–800. 35338036 10.1136/bjsports-2020-103405 PMC9252858

[81] Perna P, de Keijzer KL, Beato M. Flywheel resistance training in football: a useful rehabilitation tool for practitioners. Front Sports Act Living. 2024; 6. 10.3389/fspor.2024.1434995 PMC11257893 39036368

[82] Suarez-Arrones L, Núñez FJ, Lara-Lopez P, et al. Inertial flywheel knee- and hip-dominant hamstring strength exercises in professional soccer players: Muscle use and velocity-based (mechanical) eccentric overload. PLoS One. 2020; 15(10):e0239977. 33007010 10.1371/journal.pone.0239977 PMC7531833

[83] Ishøi L, Thorborg K, Hölmich P, et al. Sprint performance in football (soccer) players with and without a previous hamstring strain injury: an explorative cross-sectional study. Int J Sports Phys Ther. 2020; 15(6):947–957. 33344011 10.26603/ijspt20200947 PMC7727428

[84] Mendez-Villanueva A, Nuñez FJ, Lazaro-Ramirez JL, et al. Knee Flexor Eccentric Strength, Hamstring Muscle Volume and Sprinting in Elite Professional Soccer Players with a Prior Strained Hamstring. Biology (Basel). 2022; 11(1):69. 35053067 10.3390/biology11010069 PMC8773384

[85] Dijkstra HP, Pollock N, Chakraverty R, et al. Return to play in elite sport: a shared decision-making process. Br J Sports Med. 2017; 51(5):419–420. 27474390 10.1136/bjsports-2016-096209

